# Comparison of the Gut Microbiota Between *Pulsatilla* Decoction and Levofloxacin Hydrochloride Therapy on *Escherichia coli* Infection

**DOI:** 10.3389/fcimb.2020.00319

**Published:** 2020-06-30

**Authors:** Xiaoye Liu, Shangwen He, Qiuyue Li, Xiang Mu, Ge Hu, Hong Dong

**Affiliations:** ^1^Beijing Traditional Chinese Veterinary Engineering Center and Beijing Key Laboratory of Traditional Chinese Veterinary Medicine, Beijing University of Agriculture, Beijing, China; ^2^Department of Mechanics and Engineering Science, College of Engineering, Academy for Advanced Interdisciplinary Studies, and Beijing Advanced Innovation Center for Engineering Science and Emerging Technology, College of Engineering, Peking University, Beijing, China; ^3^Beijing Advanced Innovation Center for Food Nutrition and Human Health, College of Veterinary Medicine, China Agricultural University, Beijing, China

**Keywords:** *Pulsatilla* Decoction, Levofloxacin Hydrochloride, *Escherichia coli*, infection, gut microbiota

## Abstract

Gut microbiota serves as a critical indicator for gut health during treatment of pathogenic bacterial infection. Both *Pulsatilla* Decoction (abbreviated to PD, a traditional Chinese medicine compound) and Levofloxacin Hydrochloride (LVX) were known to have therapeutic effects to intestinal infectious disease. However, the changes of gut microbiota after PD or LVX treatment remain unclear. Herein, this work aimed to investigate the changes of intestinal flora after PD or LVX therapy of *Escherichia coli* infection in rats. Results revealed that PD exhibited a valid therapeutic approach for *E. coli* infection via the intestinal protection, as well as the inhibited release of IL-8 and ICAM-1. Besides, PD was beneficial to rebuild the gut microbiota via restoring *Bacteroidetes* spp in the composition of the gut microbiota. Comparatively, LVX treatment promoted the infection and ravaged gut microbiota by significantly decreasing Bacteroidetes and increasing Firmicutes. These findings not only highlight the mechanism of Chinese herbal formula, but extend the application of PD as veterinary medicine, feed additive and pre-mixing agent for improving the production of animal derived foods.

## Introduction

Gut microbiota plays a fundamental role in providing the colonization resistance of intestinal tissues against the exogenous pathogenic bacteria (Baumler and Sperandio, 2016). When it comes to infectious diseases, the problem emerged as antibiotics, especially the broad-spectrum ones cannot distinguish the intestinal beneficial bacteria from the exogenous harmful bacteria (Blaser, 2016; Lange et al., 2016). Many previous reports showed that antibiotic treatments altered the composition of intestinal microbiota, resulting in an increased risk of many other illnesses (Blaser, 2011; Angelucci et al., 2019; Dierikx et al., 2020; Zwittink et al., 2020). For instance, intestinal inflammation that is tightly linked with altered gut microbiota might be triggered by antibiotic treatment (Belkaid and Hand, 2014; Slager et al., 2014; Becattini et al., 2016). Moreover, frequent exposure of the pathogenic bacteria to antibiotics could lead to the antibiotic resistance crisis (Ventola, 2015; Yelin and Kishony, 2018). Worse still, most recent researches revealed that the drug-resistant pathogens could further promote the spread of resistant plasmid in gut and then induce secondary infection (Bakkeren et al., 2019; Wu et al., 2020).

**Figure 1:**
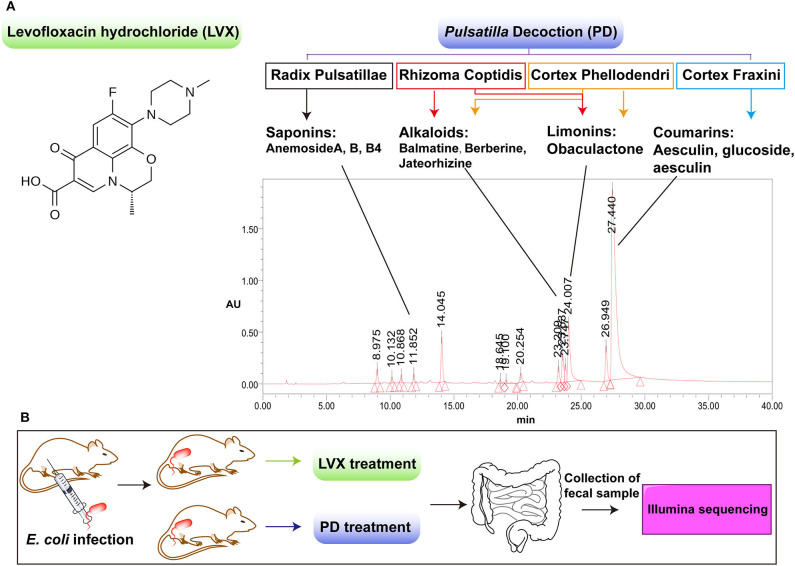
Comparison of the chemical components between Levofloxacin hydrochloride (LVX) and *Pulsatilla* Decoction (PD). **(A)** LVX belonged to one kind of quinolone antibiotics, while PD was composed by four classical herbal formula, including radix *Pulsatillae, Rhizome coptidis, Cortex phellodendri, Cortex fraxini*. The main components of PD were analyzed by LC-MS/MS analysis. **(B)** Scheme of LVX and PD treatment on *E. coli* infected rats.

Traditional Chinese medicine (TCM) includes diverse Chinese herbs with low toxicity and less resistance, which has gradually developed into a group of natural antimicrobial agents (Li et al., 2019; Huang et al., 2020). Noticeably, *Astragalus, Berberine* and many other herbs, which are compositional in many Chinese herbal formulations, have not only the antibacterial but also the anti-inflammatory effects (Auyeung et al., 2016; Ma et al., 2018; Zhang et al., 2019). *Pulsatilla* Decoction (PD) is a classic TCM compound for the treatment of heat and dysentery (Hua et al., 2020). PD consists of four classical herbs of *Radix Pulsatillae, Rhizoma Coptidis, Cortex Phellodendri*, and *Cortex Fraxini*, which contains various antibacterial and anti-inflammatory ingredients (Hu et al., 2009; Yang et al., 2018). In modern medicine, PD is also proved to have a good curative effect on bacterial diarrhea and inflammatory bowel diseases (Wang et al., 2016; Yang et al., 2018; Hua et al., 2019, 2020). As most Chinese herbal compounds were oral administration, there was a large chance for them to interact with intestinal microbes (Li et al., 2019; Huang et al., 2020). However, unlike the systemically studied inhibitory effect of PD on pathogenic bacteria in gut, little is known about the influence of PD treatment on gut microbiota. Therefore, in this paper, we carefully investigated the gut microbiota changes after antibiotic or TCM compound treatment on *Escherichia coli* induced infection (Figure 1). As shown in Figure 1A, we chose Levofloxacin Hydrochloride (LVX) as the antibiotic group, which belonged to quinolones with a broad spectrum of antibacterial effect against most Enterobacteriaceae bacteria (Perez-Pitarch et al., 2017). And PD was selected as the TCM compound group to make a comparison on the treatment of *E. coli* infection and the changes of rat intestinal microbiota. We believe our work will benefit to understand the pharmacological action of PD, and its applications of guarantee the intestinal health on the animal source food.

## Results

### PD Treatment Prevented the Intestinal Damage Induced by *Escherichia coli* Infection

**Figure 2:**
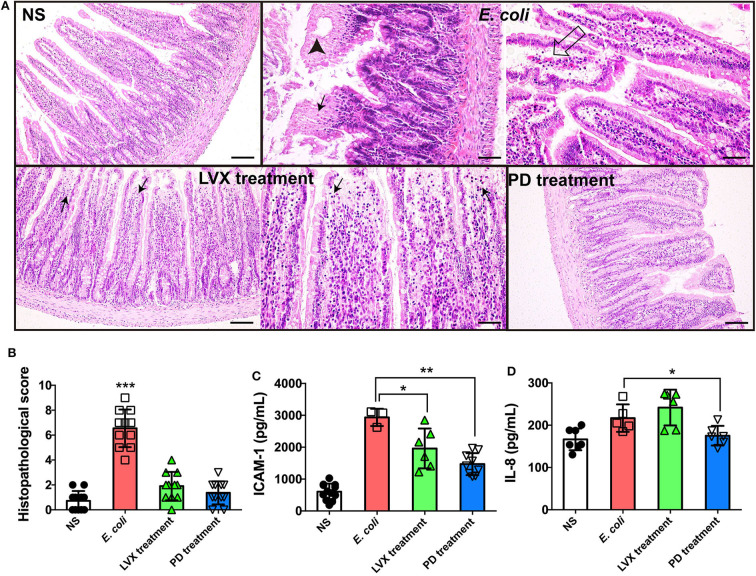
PD treatment triggered less intestinal injury comparing to LVX treatment. **(A)** HE staining of intestine under PD or LVX treatment of *E. coli* infection. Rats were infected with *E. coli* O_101_ (1 × 10^11^ cfu/kg) for 3 days. Rats treated with normal saline (NS) as an uninfected group. Then the infected rats were received oral administration of PD or LVX. Arrowhead showed coagulation necrosis, while empty arrow showed mucosa lamina propria with congestion. Black arrows pointed to focal necrosis with inflammatory cell infiltration. Scar bar = 100 μm. **(B)** The pathological scores of the intestine presented the severity of intestine pathological lesions. **(C,D)** LVX treatment increased IL-8 and ICAM-1 release. Values represented the mean ± SD (*P < 0.05, **P < 0.01, ***P < 0.001, *n* = 10).

The integrity of intestine indicated intestinal homeostasis and the health of epithelial barrier (Dupaul-Chicoine et al., 2010; Konig et al., 2016). Therefore, we firstly compared the intestinal tissue under PD or LVX treatment of *E. coli* infection by a HE staining assay. We found that *E. coli* infection led to mucosa lamina propria coagulation necrosis, focal necrosis with inflammatory cell infiltration and mucosa lamina propria with congestion (Figure 2A), suggesting that the infection was proceeding. Compared with *E. coli* treated rats, oral supplementations of LVX led to infection-mucosa lamina propria with inflammatory cellular infiltrates, while PD had almost no damage on intestinal tissue (Figures 2B,C). Additionally, in accordance with the previous study which showed that PD effectively inhibited the expression of proinflammatory cytokines including IL-1beta, IL-6, and TNF-alpha (Hu et al., 2009), PD treatment also decreased IL-8 and ICAM-1 induced by *E. coli* infection in our work (Figure 2D).

### LVX Reduced the Overall Abundances of Intestinal Microbiota

**Figure 3:**
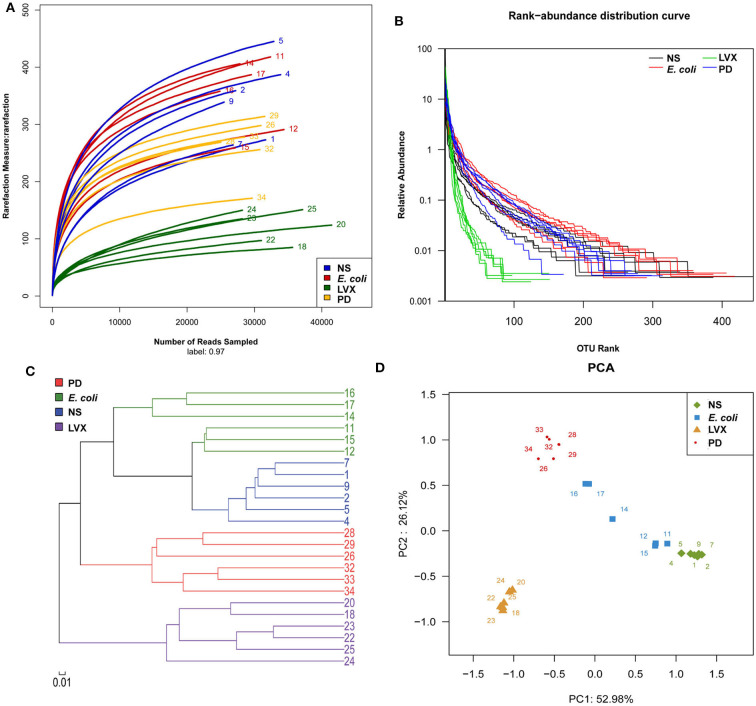
Overall structural and modulation of gut microbiota after PD or LVX treatment. **(A)** The richness and diversity of rats' fecal microbiota among four groups. Rarefaction curves were used to estimate richness (at a 97% similarity level) of rats' fecal microbiota. **(B)** Rank abundance curve was used to estimate the abundance and evenness. **(C)** Difference and similarity of microbial communities among four groups revealed by Weighted Uniface principal component analysis (PCA). **(D)** Hierarchical cluster analysis.

**Figure 4:**
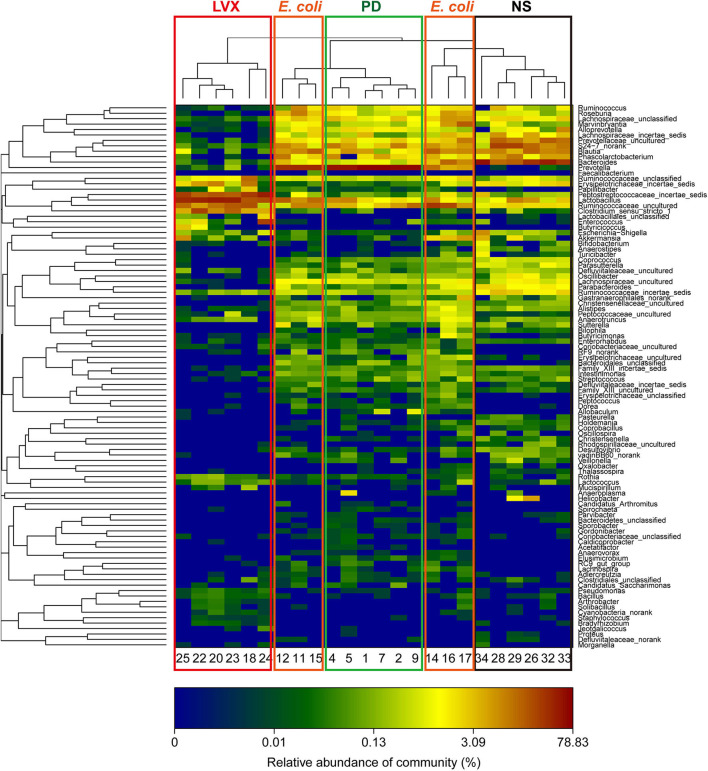
Heatmap of hierarchy cluster indicated the amount of genus in feces. Colors reflect relative abundance from low (blue) to high (red).

To analyze the changes of gut microbiota abundances under PD or LVX treatment, we firstly performed high throughput sequencing on the V3-V4 hypervariable region of bacteria *16S rRNA* gene with Illumina MiSeq to show the microbial composition. Then a total of 764838 valid reads were obtained from the 24 samples with an average of 30593 reads per sample. The good's coverage of all samples was 0.9977 ± 0.0007%, indicating that the *16S rRNA* sequences represented the majority of bacteria in the samples of this study. Rarefaction curves indicated that the most diversity of the bacteria had been covered (Figure 3A). Rand-Abundance curves showed that the abundance had been presented (Figure 3B). Weighted Unifrac principal component analysis (PCA) revealed that the gut microbiota structure changed significantly in response to different administration. Furthermore, The first principal component (PC1) distinctly separated LVX treatment from other treatments (Figure 3C). Further hierarchical cluster analysis revealed that the robust differences in LVX treatment compare to normal saline (NS) treatment and PD treatment (Figure 3D). Heatmap images also pointed out that the difference of LVX treatment from other treatments (Figure 4). Especially, PD treatment did not change the major component of intestine microbiota.

### PD Treatment Protected *Bacteroidales* spp and Did Not Facilitate the Relative Abundance of *Clostridiales* spp and *Lactobacillales* spp

**Figure 5:**
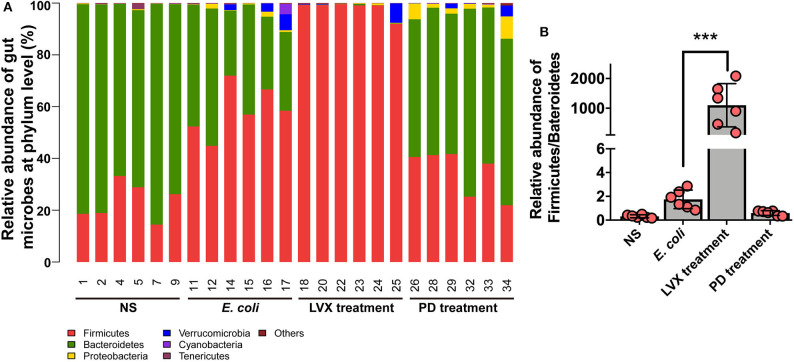
Microbial composition of PD or LVX treatment at the phylum level. **(A)** Distribution of bacterial taxa under PD or LVX treatment at the phylum level. Sequences that could not be classified into any known group were designated as “unclassified”. **(B)** Relative abundance of Firmicutes/Bacteroidetes under PD or LVX treatment. Date showed the mean ± SD (***P < 0.001, *n* = 6).

To further understand the difference of PD and LVX treatment on gut microbiota. We used the illumina sequencing assay to analyze the microbiota. We observed four major phyla including Firmicutes, Bacteroidetes, Proteobacteria, and Verrucomicrobia in all treatments (Figure 5A). Bacteroidetes abundance was mostly distributed on NS (75.85 ± 3.08) and PD (60.26 ± 2.97) treatment. While oral supplementations of LVX had dramatically decreased the relative abundance of Bacteroidetes (0.17 ± 0.08), and increased firmicutes abundance (98.06 ± 1.02). These tendencies were more obvious than those of *E. coli* infection without any treatments (Firmicutes, 58.53 ± 3.98; Bacteroidetes, 37.71 ± 4.651). The ratio of Firmicutes and Bacteroidetes population is critical for intestinal integrity (Chen et al., 2016), the downregulation of this ratio by LVX might be the main reason for the gut microbiota changes (Figure 5B).

**Figure 6:**
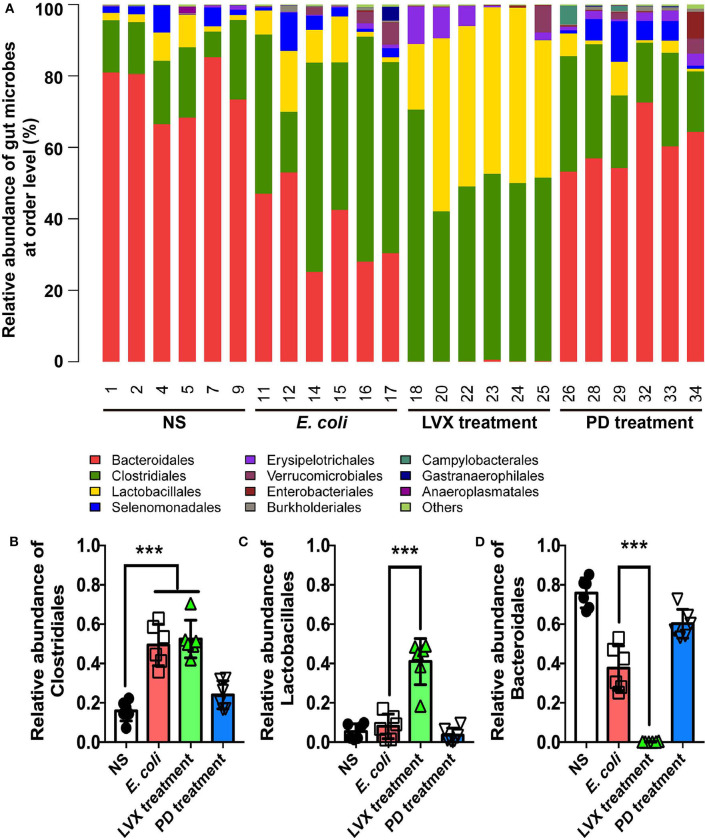
Comparison of main genera in intestine between PD and LVX treatment. **(A)** Distribution of bacterial taxa under PD or LVX treatment at the order level. **(B)** Relative abundance of *Bacteroidales, Lactobacillales*, and *Clostridiales* under PD or LVX treatment. Results showed the mean ± SD (***P < 0.001, *n* = 6).

Alternations of gut microbiota in all treatments were in an order of Bacteroidales > Clostridiales > Lactobacillales (Figure 6A). As shown, Bacteroidales was most abundant in NS (75.84 ± 3.09) and PD (60.26 ± 2.97) treatments, followed by Clostridiales (16.00 ± 2.13 and 24.09 ± 2.91), and Lactobacillales (5.40 ± 1.592) and (3.61 ± 1.46). Oral administrations of LVX had a significant impact on gut microbiota composition, indicated by that the relative abundance of Bacteroidales (0.17 ± 0.08) was nearly absent, while that of Lactobacillales (40.99 ± 4.78) and Clostridiales (52.46 ± 3.89) were increased compared with other treatments. Clostridiales was also abundant in *E. coli* infection (58.53 ± 3.98), and Lactobacillales was 1.31%, while Bacteroidetes (37.70 ± 4.65) was less than NS or PD treatment. Oral administrations of Levofloxacin hydrochloride reduced the relative abundance of Bacteroidales, but increased that of Clostridiales or Lactobacillales compared with normal and PD treatments. Intraperitoneal injection of *E. coli* also reduced the relative abundance of Bacteroidales and increased Clostridiales compared with NS and PD treatment.

We further compared the difference of microbiota distribution at the genus level in four treatment. As shown in Figures 6B–D, the proportion of Bacteroides, Prevotella and Lactobacillales were increasingly different in four treatments. Prevotella belonging to Bacteroidales was most abundant in normal treatments (66.38 ± 4.092). Bacteroides belonging to Bacteroidales was most abundant in PD treatments (32.92 ± 7.44). Lactobacillus belonging to Lactobacillales was mostly enriched in LVX treatments (35.73 ± 5.32). In contrast, Prevotella and Bacteroides were nearly absent in LVX treatment.

## Discussion

Among the intestinal microbial population, Bacteroidetes and Firmicutes accounted for 90% of the total gut microbiota (Pascale et al., 2019). Our results showed that abundances of two phylum in NS model, LVX and PD treatment were 99.22, 96.24, 98.23, 95.04%, respectively. It was evidential that a lower Bacteroidetes/ Firmicutes ratio (B/F ratio) reflected phylum-wide increase in Firmicutes and/or reduction in Bacteroidetes, which were related to many diseases, such as obesity (Pascale et al., 2019), colon cancer (Zhu et al., 2014) and immunosuppression (Xu and Zhang, 2015). B/F ratio might also serve as an index to evaluate the imbalance of gut microbiota from various illnesses. In our study, we confirmed *E. coli* infection can disrupt the B/F ratio. Treatment with PD resulted in a considerable increase in the relative abundance of Bacteriodetes and the B/F ratio (1.732), comparing to the *E. coli* group (0.644) or antibiotic Levofloxacin hydrochloride groups (0.002). Furthermore, the B/F ratio of 3.245 in normal rats was consistent with the previous researches (Gu et al., 2013). Previous studies had also suggested that the imbalance in the ratio of intestinal bacteria played a vital role in the development of obesity with induction of systemic inflammation, and acceleration of fat deposition (John and Mullin, 2016). *E. coli* colonized in GI tract could release toxins and inflammation cytokines, causing breakdown of the tight junctional complexes between the intestinal epithelial cells and disruption channel regulation. They led to an influx of water and ions into the intestinal lumen, causing infection (Viggiano et al., 2015). Our study suggested that oral administration of PD significantly decreased the level of proinflammatory IL-8, ICAM-1 and improved intestinal histopathology injurie in comparison with the *E. coli* group. Although antibiotic Levofloxacin hydrochloride also decreased the level of proinflammatory ICAM-1 and improved intestinal histopathology injurie in comparison with the *E. coli* group, the level of IL-8 was elevated. After replenishing PD, the levels of serum proinflammatory IL-8 and intestinal histopathology injurie recovered to the normal level in the *E. coli*-infected group, indicating that the symptoms of intestine were improved. As a broad-spectrum antibiotic, Levofloxacin hydrochloride treatment broadly killed bacterial populations, such as Bacteroidetes, leading to the disturbed intestinal microbiota. Therefore, the corresponding effect of treating infection was inferior to that of PD. In the previous study, the reduced Bacteroidetes and Firmicutes, combined with the increased Proteobacteria caused antibiotic-induced imbalances in gut microbiota, which aggravated cholesterol accumulation and liver injuries in rats fed with a high-cholesterol diet (Hu et al., 2015). Bacteroidetes was known to promote the catabolism of plant cell wall (Spence et al., 2006) and the increased abundance of Bacteroidetes might ameliorate the intestinal mucosal barrier function and ultimately enhanced the innate immune responses (Sonnenburg et al., 2005). These results indicated that PD, which could regulate the distribution of the intestinal flora through increasing and/or restoring the B/F ratio, played an essential role in combating *E. coli*-induced infection.

At order level, the compositions of four bacterial populations were dominated by three orders: Lactobacillales (L), Clostridiales (C), Bacteroidales (B). Lactobacillales and Clostridiales belonged to Firmicutes. Bacteroidales belonged to Bacteroidetes. The major difference lied in the ratio of Bacteroidales /Lactobacillales+ Clostridiale: B/L+C (normal groups) = 3.54, B/L+C (model groups) = 0.65, B/L+C (LH groups) = 0.001, B/L+C (PD groups) = 2.17, which was consistent with the results at phylum level. Antibiotics also broadly reduced Bacteroidales and increased Lactobacillales and Clostridiales compared with normal rats. Obviously, Clostridiales was highly presented in both model and antibiotic group. Treatment with *Pulsatilla* Decoction could reduce the relative abundance of Clostridiales*. Clostridium difficile* (Abu Faddan et al., 2016), a species belonging to order Clostridiales, produced multiple toxins which might produce infection and inflammation. These results suggested that *Pulsatilla* Decoction could modulate the distribution of the intestinal flora in order level to alleviate infection. In fact, Chinese herbal medicine compound has no direct antibacterial effect *in vitro* but enhances the antibacterial effect *in vivo* by mobilizing innate immunity. As report showed, among 30 Chinese herbs, only Scutellaria barbata had the 100% antibacterial activity *in vitro* (Tsai et al., 2018). Actually, Chinese herbs, such as Astragali Radix are benefit for intestinal bacterial conversion (Zhou et al., 2012). Both Chinese herbs and the gut microbiota had the regulation of innate immunity (Wójcik et al., 2009; Wang et al., 2019). Therefore, Chinese herbs rarely has a direct antibacterial ability, but it can rely on inhibiting bacterial growth proteins to inhibit infection (Si et al., 2016) or enhance the regulatory factors that promote innate immunity to achieve antibacterial effect (Ma et al., 2013, 2018; Si et al., 2016; Wang et al., 2016; Yang et al., 2018; Pascale et al., 2019). Consisting with the long-term research of our labs that the integrity of barrier cells such as epithelial cells and endothelial cells contributed to the bactericidal function of immune cells such as neutrophils (Liu et al., 2016). The traditional Chinese medicine compound promotes the innate immune and antibacterial effect by protecting the integrity of such barrier cells.

## Conclusion

Our work compared the difference on gut microbiota in *E. coli* infected rats after PD or LVX treatments. PD protected the intestinal tissue and regulated the balance of gut microbiota via restoring the composition of the gut microbiota in *Bacteroidetes* spp. In contrast, LVX treatment leaded the intestinal tissue damage, as well as ravaged gut microbiota by significantly decreasing Bacteroidetes and increasing Firmicutes. This work provided experimental data for the study of the mechanism of Chinese medicine prescription in the treatment of bacterial infection. It was also emphasized that the important role of intestinal flora in the prevention and therapy of bacterial infection.

## Methods and Materials

### Animals

Specific pathogen-free male Sprague Dawley Rat (190–210 g) were supplied by Vital River Laboratory Animal Technology (Beijing, China). Rats were kept at a temperature of 22 °C and 12-h light/dark cycle environment for at least 1 week before use, and fed on the same batch of standard laboratory diet to minimize the variation of environmental factors. The present study was approved by the Institutional Animal Care and Use Committee of the Academy of Military Medical Sciences (Beijing, China; approval no. SYXK2014-0002). All animal care and experimental procedures were conducted according to the Chinese Laboratory Animals' Welfare and Ethics guidelines.

### Preparation of *Pulsatilla* Decoction (PD) Extract Powder

PD consisted of four herbs including Radix Pulsatillae (60 g), Rhizoma Coptidis (30 g), Cortex Phellodendri (45 g), and Cortex Fraxini (60 g). All those herbs were purchased from Tong Ren Tang Medicinal Materials Company (Beijing, China), which was authenticated by professor Pengyue Li from Beijing university of Chinese medicine. Voucher specimens with specific storage code were well-deposited at Beijing university of Chinese medicine. PD were extracted twice with boiling water (1:10 and then 1:8, w/v) for 1 h. The water extracts were combined, concentrated in vacuum into 2 g/mL, and then stored at 4°C until use. Chromatographic fingerprint analysis on PD could be found in our previous study.

### Infection and Therapy

Rats were infected by intraperitoneal injection of *E. coli* O_101_ (China Institute of Veterinary Drugs Control, O101: K91, K88, 1 × 10^11^ cfu/kg) for three consecutive days. Meanwhile, 10 rats were randomly selected for each group, which were subject to the treatment with isovolumetric normal saline (NS) as negative control, oral absorption of PD (7.5 g/kg) or levofloxacin hydrochloride (LVX, Sangjing Pharmachceutical co., LTD, 100 mg/kg, mimicing the human dose of 500 mg/day), respectively. Another three consecutive days were employed as therapeutic schedule of infected rats, respectively. Blood samples were collected before sacrifice. Serums of rats were selected by centrifugation at 3,000 r/min for 10 min at 4°C. Fresh fecal samples were collected at the day of sacrifice and then kept at −80°C.

### Histopathological Analysis

Intestine samples were fixed in 4% paraformaldehyde. The jejunum of small intestine was selected prepared for hematoxylin and eosin staining (HE staining). Olympus microscope (Olympus Optical Co., Ltd.) was used to observe and score the histopathological changes as previous methods (Han et al., 2014). Each group contained 10 rats and all samples for pathological analysis were blindly selected.

### ELISA

Cytokines IL-8 and ICAM-1 (ENZO life sciences) were detected by enzyme-linked immunosorbent assay (ELISA) according to the manufacturer's instructions.

### DNA Extraction, PCR Amplification and Illumina Sequencing

After PD and LVX treatments for 3 days, fresh fecal samples from random selected 6 rats were collected individually. Then these samples were frozen immediately in liquid nitrogen and stored at −80°C for further analysis. Total DNA was extracted from fecal samples by using the E.Z.N.A^.®^ Soil DNA Kit (Omega Bio-tek, Norcross, GA, USA) according to the manufacturer's protocol. The variable regions V3-V4 of the 16S rRNA were amplified. Equimolar concentrations of purified PCR products were pooled and paired-end sequenced (2 × 300 bp) on an Illumina MiSeq platform (Illumina Inc., San Diego, CA, USA).

Sequences data were subject to bioinformatic analysis. Operational taxonomic Units (OTUs) were using UPARSE software (version 7.1) (http://drive5.com/uparse/). The 16S rRNA sequences of *E. coli* O_101_ to compare and distinguish foreign *E. coli* from the local *E. coli* in gut. OTUs that reached 97% similarity were used for Good's coverage and rarefaction curve analysis (Schloss et al., 2011). Through the classification operation, the sequences were divided into many groups according to their similarities, and a group was an OTU. According to different similarity levels, all sequences could be divided into OTUs, and biological information statistical analysis was usually performed on OTUs at 97% similarity levels. Community structure comparisons with Principal Component Analysis (PCA) were based on weighted UniFrac distance. Hierarchical cluster analysis, Rank-Abundance and heatmap were generated according to R software package (http://www.R-project.org) (Wang et al., 2012).

### Statistical Analysis

Data were expressed as means ± standard deviation (SD) and analyzed by using GraphPad Prism 8 software. The criterion of significance was conducted using unpaired *t*-test by one-way ANOVA. At least 10 rats were involved in every group for animal experiments. All experiments were performed on no <3 biological replicates. All animals were used for analysis unless the mice died.

## Data Availability Statement

The datasets generated for this study are available on request to the corresponding author.

## Ethics Statement

The experimental protocols and all animals were approved by the Genentech Institutional Animal Care and Use Committee at the Beijing University of Agriculture (SYXK, 2015-0004) and China Agricultural University (SYXK, 2016-0008).

## Author Contributions

HD conceived the project. XL and HD did for the research design. XL, SH, and QL performed the experiments. XL, SH, GH, and HD did data analysis. GH and XM carried out the histopathological analysis. XL, GH, and HD wrote the manuscript. All authors read and approved the manuscript.

## Conflict of Interest

The authors declare that the research was conducted in the absence of any commercial or financial relationships that could be construed as a potential conflict of interest.

## Abbreviations

*E. coli*: *Escherichia coli*
PD: *Pulsatilla* Decoction
LVX: Levofloxacin Hydrochloride.
